# The Living Conditions of Children with Shared Residence – the Swedish Example

**DOI:** 10.1007/s12187-017-9443-1

**Published:** 2017-01-17

**Authors:** Emma Fransson, Sara Brolin Låftman, Viveca Östberg, Anders Hjern, Malin Bergström

**Affiliations:** 1 Centre for Health Equity Studies, CHESS, Stockholm University/Karolinska Institutet, SE-10691 Stockholm, Sweden; 2 Department of Medicine, Clinical Epidemiology Unit, Karolinska Institutet, SE-17177 Stockholm, Sweden

**Keywords:** Divorce, Shared parenting, Child health, Family policy, Joint physical custody, Welfare

## Abstract

Among children with separated parents, shared residence – i.e., joint physical custody where the child is sharing his or her time equally between two custodial parents’ homes – is increasing in many Western countries and is particularly common in Sweden. The overall level of living among children in Sweden is high; however, the potential structural differences between children in various post-separation family arrangements have not been sufficiently studied. Potential risks for children with shared residence relate to the daily hassles and stress when having two homes. This study aims at investigating the living conditions of children with shared residence compared with children living with two custodial parents in the same household and those living with one custodial parent, respectively. Swedish national survey data collected from children aged 10–18 years (n ≈ 5000) and their parents were used. The outcomes were grouped into: Economic and material conditions, Social relations with parents and peers, Health and health behaviors, Working conditions and safety in school and in the neighborhood, and Culture and leisure time activities. Results from a series of linear probability models showed that most outcomes were similar for children with shared residence and those living with two custodial parents in the same household, while several outcomes were worse for children living with one parent. However, few differences due to living arrangements were found regarding school conditions. This study highlights the inequalities in the living conditions of Swedish children, with those living with one parent having fewer resources compared with other children.

## Introduction

**Fig. 1:**
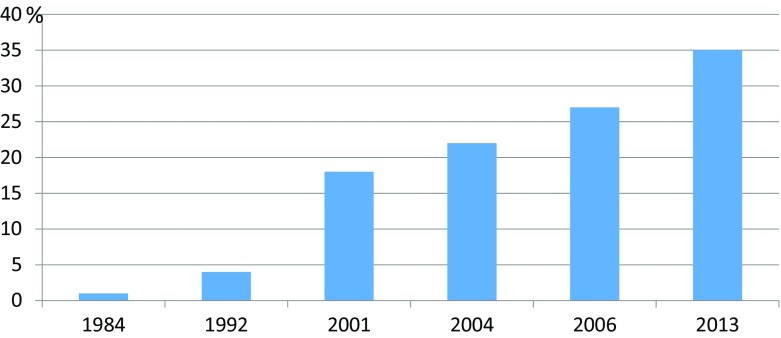
Percentage of children in Sweden with separated parents, with shared residence. (Source: Swedish Government official report 2011; Statistics Sweden 2014)

Shared residence, i.e., joint physical custody where the child is sharing his or her time equally between two custodial parents’ homes, is increasing in many Western countries and is common in parts of Northern Europe, particularly in Sweden. Traditionally, after parental separation, children have continued to live with their mother. However, during the past few decades, this picture has changed; thus, shared residence is almost as common as the traditional sole mother care among children with separated parents in Sweden (Statistics Sweden 2014; Swedish Government Official Report 2011, see also Fig. 1). Parental separation has been associated with lower wellbeing in both parents and children (Amato 2000; Berkman et al. 2015; Weitoft et al. 2004). However, growing evidence has suggested that adolescents with shared residence fare better than those in sole parent care (Bergström et al. 2015; Nielsen 2014). Most of the studies in the field have failed to identify the mechanisms involved for children in the different living arrangements. Socioeconomic and other parental factors, however, seem to account for part of the difference in child health outcomes (Bergström et al. 2014; Bjarnason et al. 2012), suggesting that some variance could be attributed to economic standards as well as to the wellbeing of the parents and the quality of the parent-child relationships. Despite the growing number of studies regarding the health of children in different living arrangements, knowledge concerning the extent to which children’s living conditions differ between the living arrangements, in a broader sense, is lacking. Using data from the yearly Swedish Living Conditions Survey (ULF) and its child supplement (Child-ULF), this study aims at elucidating the potential differences in living conditions among children and adolescents with different living arrangements.

### Swedish Family Policies

Sweden has long had a tradition of family policies that acknowledge both mothers and fathers as supposedly being engaged in paid work as well as in caring for children. Since 1974, both mothers and fathers have had the possibility to use the paid parental leave policy. In 2012, the share of days used by fathers comprised 24% (Social Insurance Report 2013). Swedish public policies are also translated into a relatively high involvement in child care among Swedish fathers (Plantin et al. 2011). Accordingly, Swedish parents often share the daily parental responsibility for children also after a divorce or separation (The Swedish Government Offices 1999). Since 1998, the Swedish court has also been able to decide on shared residence when one parent opposes, if the court still finds the solution to be in the best interest of the child (The Swedish Government Offices 1997). Shared residence, however, is a less common post-separation arrangement among families with non-Swedish background (Bergström et al. 2013) and among those in the lowest income category (Swedish Government Official Report 2011).

### The Welfare of Children in Sweden

From an international perspective, children in Sweden fare well. In the index of child wellbeing in Europe, developed by Bradshaw and Richardson (2009), Sweden is rated as the second highest (after the Netherlands) of the 29 included countries and also scores highly in the specific domains such as child health, personal relationships, risk and safety, and housing and environment. Despite the generally high standard of living, there are nevertheless areas where problems are more common (e.g., subjective health complaints, see (Inchley et al. 2016)). There are also areas involving systematic variation between groups of children. More specifically, children living with a single parent and children of immigrants tend to report poorer resources compared with their peers living with two custodial parents and those of Swedish-born parents, respectively (Jonsson and Östberg 2010). Moreover, these categories are over-represented among children who live in absolute poverty, in terms of low income standard (Mood and Jonsson 2014). It has been shown that it is not only children who live with a single parent but also those in other post-separation living arrangements that have less beneficial living conditions. For instance, it has been found that children living with a single parent and those living with a parent and a step-parent are more likely to report psychosomatic health complaints than their peers who live with two custodial parents (Låftman and Östberg 2006). They have also been identified as being more likely to suffer from material and economic deprivation and to have problems with participation and consumption on par with their peers (Mood and Jonsson 2015). Thus, family structure constitutes a central inequality dimension among children in Sweden, with a systematic gap between those living with two custodial parents in the same household and those with other living arrangements. Nevertheless, empirical research is still limited on the potential differences between children in various post-separation family arrangements where children with shared residence have been distinguished as a separate category.

### Potential Drawbacks of Shared Residence

Concerns have been raised regarding the potential stress for children living in two homes and in two family cultures (Gilmore 2006; McIntosh et al. 2011). Other concerns relate to the potential difficulties in maintaining social contacts when moving between two neighborhoods (Prazen et al. 2011). In an interview study with adolescents, the obvious drawbacks of shared residence have been described, for example, as logistics such as travelling between the homes and lacking one’s personal items (Cashmore et al. 2010). Other worries that have been highlighted, for example, in social media include the risk for children becoming spoiled when having two homes (Avitable 2010), while child professionals have pointed at the risk for the child being more exposed to parental conflict and of feeling torn between parents (Buchanan et al. 1991; Gilmore 2006). For young children, the debate has mostly regarded the potential risk of being separated from the mother (McIntosh et al. 2011), while others have emphasized the importance of the continued involvement of both parents on an everyday basis (Nielsen 2013; Warshak 2014). Furthermore, parental monitoring of children could be expected to be lower in post-separation living arrangements. Children with shared residence might be less monitored if the parents fail to communicate. Low parental monitoring has been associated with an increased risk of mental ill-health in youth (Fröjd et al. 2007).

Despite the increasing proportions of children having shared residence in Sweden, knowledge about the living conditions of this group still remains limited. To date, empirical studies of children with shared residence have largely focused on health-related outcomes and parental relations, but a picture of their level of living in a broader sense is lacking, particularly a picture that is grounded in data including not only children’s own reports about their living conditions but also reliable measures of household characteristics such as parental education.

### Aim and Research Questions

The overall aim of this study was to provide a thorough description of the living conditions of children with shared residence and to compare the living conditions of this group to those living with two custodial parents in the same household and to those living with one custodial parent. To do this, we used Swedish national survey data including information collected from both children and their parents. This study investigates the potential differences due to children’s living arrangements with regard to economic and material conditions, social relationships, health and health behaviors, conditions in school and neighborhood, as well as culture and leisure time activities. In order to (at least partially) control for the selection into different living arrangements, the analyses are adjusted for factors previously known to differ between parents with shared residence and with sole parental care, namely, the level of education (Statistics Sweden 2014) and non-Swedish background (Bergström et al. 2013).

## Data and Method

### Data Material

The data were derived from the Swedish Living Conditions Survey (ULF) and the Living Conditions Survey of Children (Child-ULF) (see http://www.scb.se/LE0101-en/). The design of the data makes it possible to link the information collected from the parents to the information collected from the children. We used pooled data from the survey years 2007–2011. Both ULF and Child-ULF are conducted by telephone and carried out by Statistics Sweden. ULF is based on a nationally representative sample of individuals living in Sweden aged 16 years and older. The interview covers a wide range of living conditions, including education, occupation and employment, family relations, and health. For the survey years 2007–2011, the total non-response rate was 27–41% annually, with an overrepresentation of persons born outside of Sweden, single parents (compared with parents living with a partner), individuals with a low level of education, those having a lower income, and recipients of social assistance (Statistics Sweden 2016). All children between 10 and 18 years who live in the adult respondents’ household at least half the time comprise the sample frame of Child-ULF. Similar to the interview with the adult respondents, the child supplement includes questions covering a broad range of living conditions such as children’s own financial resources, relations with parents and peers, health and health-related behaviors, and education and working conditions in school. The rate of non-responding children, calculated among those whose parents agreed to take part in the survey, was between 26 and 37% during the years 2007–2011. Although there is not yet any available detailed analysis of the non-response of Child-ULF, it is likely that there was systematic bias also among the responding children in the sample frame. In a similar survey, Child-LNU 2000, the non-response was higher among, e.g., 18-year-olds, those not living with two custodial parents, and those with foreign-born parents (Jonsson et al. 2001). In the present paper, the analytic sample included about 5000 children 10–18 years of age, with some variation for different outcomes.

### Variables

#### Independent Variable

The *living arrangement* categories were based on data from the adult survey about the child’s residency. Children living less than half the time with the adult participant were not included in the sample frame of Child-ULF. For the children included, the parent answered the following questions regarding child residency: “Does the child live with you all of the time or part of the time” with the response alternatives “all or nearly all of the time” or “part of the time.” If the parent answered “part of the time,” new response alternatives were “half of the time ‘shared residence,’” or “more than half of the time.” The parent also provided information on whether or not the child’s other parent lived in the same household. For purposes of this study, the categories used in the analyses are: *Household with two custodial parents*; *Shared residence*, i.e., children who live with two custodial parents approximately half the time in each parent’s home; and *Household with one custodial parent*. For the two latter categories, the homes could also include a step-parent. Children living more than half of the time but not full time with one custodial parent (*n* = 104) were excluded to make a clear cut between the groups. Moreover, children in foster care (*n* = 12) and children with missing data on any of the background variables (*n* = 39) were excluded.

#### Control Variables

*Child’s gender* and *age* were used as control variables. Age was divided into three groups: 10–12, 13–15, 16–18 years of age.

*Parental education* was constructed from the information on the responding parent’s level of education, obtained from the adult ULF survey and classified into three categories. A low level of education was equivalent to any level less than three years of senior high school. A medium level of education was equivalent to three years of high school but less than three years of graduate school. A high level of education was equivalent to at least three years of university studies.

*Parental country of birth* was based on information from the Register of the Total Population and coded as “Sweden” or “Other,” with the latter group being comprised of children with two parents born outside Sweden. For children of separated parents, the categorization was based on the available parent’s birth country. For a few cases (*n* = 6) where the information from the custodial parent was lacking, the step-parent’s origin was used instead.

#### Dependent Variables

The dependent variables are briefly presented below. For details about the construction of these variables, see Appendix 1.

Four variables regarding economic and material conditions were used: *Having an own room, Cash margin*, *Cannot buy same things as friends,* and *Cannot afford to join friends*.

Five variables were used to measure social relations with parents: *Gets on well with mother, Gets on well with father, Mother has time for me, Father has time for me,* and *My parents know most of my friends’ parents*. Five variables were used to measure social relations with peers: *Have at least one close friend in class*, *Bring friends home weekly*, *Visit friends in their home weekly*, *Bullied at school,* and *Bullied on the Internet*.

Four variables were used to measure health: *Self-rated health (less than good)*, *Psychological complaints*, *Psychosomatic complaints,* and *Stress*. Four variables were used to measure health behaviors: *Smoking weekly*, *Alcohol use at least every other week*, *Exercise weekly, and Skipping breakfast weekly*.

Five variables were used to measure working conditions and safety in school: *Too high pace at school*, *Lack of order in classroom*, *Teachers help in school when needed*, *I do better at school than most others*, and *Feeling unsafe during breaks at school*. Two variables were used to measure safety in the neighborhood: *Been threatened, hit, or chased in my neighborhood* and *Feeling unsafe in the neighborhood*.

Four variables were used for weekly leisure time activities: *Read books weekly*, *Organized sports activity weekly*, *Organized non-sport activity weekly*, and *Do housework ≥ 3 h weekly*. Three variables were used for cultural experiences during the past six months: *Theater*, *Cinema,* and *Museum*.

### Ethics

Ethical permission for the study has been provided by the Regional Ethical Review Board of Stockholm (dnr 2012/1184–31/5).

### Statistical Methods

Since the children were sampled through the adult respondents, the sampling probabilities differed between children. For instance, children living with two parents were more likely to be sampled than children living with one parent, and children with shared residence whose parents had re-partnered could be sampled through up to four adults (i.e., custodial parents and step-parents). Thus, a sampling weight based on the number of adults the child lived with was used in the descriptive analyses. Since it has been pointed out that it is problematic to compare odds ratios from logistic regressions between models with different independent variables, we conducted linear probability models (LPM), i.e., linear regression analyses of dichotomous outcomes, where the coefficients can be interpreted in terms of percentage units (Mood 2010). All analyses were computed using Stata 13. To adjust for the fact that the observations were not independent, with some children (i.e., siblings and step-siblings) living in the same households, we used Stata’s robust cluster command to obtain robust standard errors. The regressions were modeled in two steps: the first model (“Crude”) was adjusted for the child’s gender and age group as well as survey year, and the second model (“Adjusted”) was additionally adjusted for parental education and country of birth.

## Results

Descriptive characteristics of the data, by living arrangement groups, are provided in Table 1. The proportions of boys and girls were quite evenly distributed across the living arrangement groups, while age was not. Older teens, 16–18 years, were more often living with one parent, and 13–15-year-olds were the most common age group with shared residence. High education was more common among parents in households with two custodial parents and with shared residence, while low education was common in households with just one custodial parent. In the category ‘shared residence,’ most parents had Swedish background (94.6%), and in households with one custodial parent, the reported proportion was 83.6%.

**Table 1:** Descriptive characteristics of the data, by living arrangement. Unweighted percent (n within brackets). *N* = 5125

	Household with two custodial parents	Shared residence	Household with one custodial parent	All
Gender				
Boys	49.2 (1857)	50.7 (252)	47.3 (404)	49.0 (2513)
Girls	50.8 (1917)	49.3 (245)	52.7 (450)	51.0 (2612)
Age group				
10–12	33.3 (1256)	32.6 (162)	23.0 (196)	31.5 (1614)
13–15	34.6 (1306)	37.2 (185)	32.7 (279)	34.5 (1770)
16–18	32.1 (1212)	30.2 (150)	44.4 (379)	34.0 (1741)
Parental education				
Low	39.1 (1474)	38.0 (189)	46.7 (399)	40.2 (2062)
Medium	34.4 (1299)	36.4 (181)	34.9 (298)	34.7 (1778)
High	26.5 (1001)	25.6 (127)	18.4 (157)	25.1 (1285)
Parental country of birth				
Sweden	90.2 (3403)	94.6 (470)	83.6 (714)	89.5 (4587)
Other	9.8 (371)	5.4 (27)	16.4 (140)	10.5 (538)
All	73.6 (3774)	9.7 (497)	16.7 (854)	100.0 (5125)

### Economic and Material Conditions

Differences in material conditions and economy with regard to family living arrangements are presented in Table 2. The only difference found between children living with two parents in the same household and those with shared residence is that the latter group more often reported not being able to afford to join friends for activities. For children living with one parent, in contrast, all the studied economic and material conditions were shown to be worse, compared with children living with two parents. Children in households with one parent also reported less resources, compared with children with shared residence with respect to having a higher probability of not having an own room, not being able to provide cash when needed, as well as not being able to afford to buy the same things as peers, and not being able to afford to join friends. However, when controlling for parental education and country of birth, there was no longer a difference in the probability of not having an own room.

**Table 2:** Economic and material conditions. Weighted percent and coefficients from linear probability models (LPM). *n* = 5075–5124

	Household with two custodial parents (ref.)	Shared residence	Household with one custodial parent	Sig. diff. Shared residence vs. One custodial parent
Own room^a^				
%	95.0	93.3	89.1	
Crude^b^	0.00	-0.01	-0.05***	**
Adjusted^c^	0.00	-0.02	-0.04**	n.s.
Cash margin				
%	90.8	88.1	86.2	
Crude^b^	0.00	-0.02	-0.07***	*
Adjusted^c^	0.00	-0.02	-0.06***	*
Cannot buy same things as friends				
%	16.7	19.6	27.1	
Crude^b^	0.00	0.03	0.10***	**
Adjusted^c^	0.00	0.03	0.10***	**
Cannot afford to join friends				
%	10.1	14.0	21.4	
Crude^b^	0.00	0.04*	0.10***	**
Adjusted^c^	0.00	0.04*	0.10***	*

^a^During 2007–2008, all respondents were posed the question whether or not they had their own room. During 2009–2011, children with joint physical custody were asked to specify whether they had their own room in their mother and father’s home, respectively. Children who responded that they had their own room in either one or both parents’ homes were coded as having their own room

^b^Adjusted for gender, age group, and survey year

^c^Adjusted for gender, age group, parental education, parental country of birth, and survey year

***p < 0.001 **p < 0.01 *p < 0.05

### Social Relations

Table 3 presents the differences in relations with parents and peers by family living arrangements. Compared with children living with two parents in the same household, children with shared residence reported more often that their father had time for them. On the other hand, children with shared residence reported less often that parents knew most of their friends’ parents, compared with children living in one household with two parents. Regarding peer relations, there were practically no differences between children with shared residence and those with two parents in one household. When comparing children living with two parents with those living with one parent, all the studied measures of social relations differed between the two categories, except for visit friends in their home weekly, which was not significant. The clearest differences found between children with shared residence and those living with one parent regarded relations with parents. Children living with one parent were more likely to report not getting on well with their parents, and less likely to claim that the parents had time for them and that they knew the parents of their friends. Most aspects of peer relations were found to be worse for children living with one parent compared to those with shared residence, including having a close friend in class, visit friends in their home weekly, and being exposed to bullying at school as well as on the Internet; however, several of these associations turned non-significant in the adjusted analyses.

**Table 3:** Social relations with parents and peers. Weighted percent and coefficients from linear probability models (LPM). *n* = 4970–5125

	Household with two custodial parents (ref.)	Shared residence	Household with one custodial parent	Sig. diff. Shared residence vs. One custodial parent
Gets on well with mother				
%	94.1	94.0	88.8	
Crude^a^	0.00	0.00	-0.05***	**
Adjusted^b^	0.00	0.00	-0.05***	**
Gets on well with father				
%	93.5	90.6	79.3	
Crude^a^	0.00	-0.03	-0.14***	***
Adjusted^b^	0.00	-0.03	-0.14***	***
Mother has time for me				
%	94.9	95.6	90.9	
Crude^a^	0.00	0.01	-0.05***	***
Adjusted^b^	0.00	0.01	-0.05***	***
Father has time for me				
%	87.2	92.5	76.0	
Crude^a^	0.00	0.05***	-0.11***	***
Adjusted^b^	0.00	0.05***	-0.11***	***
My parents know most of my friends’ parents				
%	61.5	53.5	41.6	
Crude^a^	0.00	-0.08**	-0.16***	**
Adjusted^b^	0.00	-0.09***	-0.16***	*
Has at least one close friend in class				
%	94.6	94.7	90.1	
Crude^a^	0.00	0.00	-0.03**	*
Adjusted^b^	0.00	0.00	-0.03**	*
Brought friends home weekly				
%	78.9	76.8	71.0	
Crude^a^	0.00	-0.02	-0.06**	n.s.
Adjusted^b^	0.00	-0.02	-0.06**	n.s.
Visited friends in their home weekly				
%	83.4	85.9	80.9	
Crude^a^	0.00	0.03	-0.02	*
Adjusted^b^	0.00	0.02	-0.01	n.s.
Bullied at school				
%	7.5	9.6	12.0	
Crude^a^	0.00	0.02	0.06***	*
Adjusted^b^	0.00	0.02	0.05***	n.s.
Bullied on the Internet, at least once^c^				
%	6.6	6.5	11.0	
Crude^a^	0.00	-0.01	0.04*	*
Adjusted^b^	0.00	-0.01	0.04*	*

^a^Adjusted for gender, age group, and survey year

^b^Adjusted for gender, age group, parental education, parental country of birth, and survey year

^c^Question posed only during 2009–2011 (*n* = 2496)

***p < 0.001 **p < 0.01 *p < 0.05

### Health and Health Related Behaviors

With regard to health and health related behaviors (see Table 4), there were practically no statistically significant differences between children with two parents in one household and those with shared residence, the exception being that those with shared residence were more likely to skip breakfast. In contrast, all the studied outcomes differed between children living with two parents and those living with one parent. Some differences were also found between children with shared residence and those living with one parent. Children in the latter group were more likely to report less than good self-rated health and more psychosomatic complaints, being stressed as well as to smoke and to skip breakfast, also in the adjusted analyses. Children living with one parent were less likely to exercise on a weekly basis than those with shared residence and more likely to report being stressed. This latter association, however, became non-significant in the adjusted model. However, no significant differences were found between these two categories for psychological complaints or alcohol use.

**Table 4:** Health and health behaviors. Weighted percent and coefficients from linear probability models (LPM). *n* = 5111–5118

	Household with two custodial parents (ref.)	Shared residence	Household with one custodial parent	Sig. diff. Shared residence vs. One custodial parent
Self-rated health (less than good)^a^				
%	12.4	13.3	26.9	
Crude^b^	0.00	0.01	0.13***	***
Adjusted^c^	0.00	0.01	0.13***	***
Psychological complaints				
%	9.1	12.2	15.4	
Crude^b^	0.00	0.02	0.06***	n.s.
Adjusted^c^	0.00	0.03	0.05***	n.s.
Psychosomatic complaints				
%	17.4	19.6	26.8	
Crude^b^	0.00	0.02	0.08***	*
Adjusted^c^	0.00	0.02	0.08***	*
Stressed (more than weekly)				
%	16.4	16.2	24.7	
Crude^b^	0.00	0.00	0.05**	*
Adjusted^c^	0.00	0.01	0.05**	*
Smoking weekly^d^				
%	5.3	6.7	17.7	
Crude^b^	0.00	0.01	0.11***	***
Adjusted^c^	0.00	0.01	0.10***	***
Alcohol use at least every other week^e^				
%	9.5	10.9	15.0	
Crude^b^	0.00	0.02	0.04**	n.s.
Adjusted^c^	0.00	0.01	0.05**	n.s.
Exercise weekly				
%	65.8	66.3	58.8	
Crude^b^	0.00	0.00	-0.06**	*
Adjusted^c^	0.00	0.00	-0.05**	n.s.
Skipping breakfast weekly				
%	12.9	16.0	26.7	
Crude^b^	0.00	0.03	0.11***	**
Adjusted^c^	0.00	0.04*	0.10***	**

^a^Question posed only during 2009–2011 (*n* = 2841)

^b^Adjusted for gender, age group, and survey year

^c^Adjusted for gender, age group, parental education, parental country of birth, and survey year

^d^Question posed to 10–18-year-olds during 2007–2008, but only to 13–18-year-olds during 2009–2011 (*n* = 4223)

^e^Question posed only to 13–18-year-olds (*n* = 3373)

***p < 0.001 **p < 0.01 *p < 0.05

### Working Conditions and Safety in School and in the Neighborhood

The conditions in school as well as in the neighborhood due to living arrangement are displayed in Table 5. Overall, no statistically significant differences were found between children living with two parents in one household and those with shared residence. In the comparison between children with two custodial parents and those with one, it is seen that those living with one parent assessed their school performance as being lower in relation to their peers. In addition, children living with one parent reported feeling unsafe during breaks at school, having been threatened, hit or chased, and feeling unsafe in the neighborhood, more often. Children living with one parent reported feeling unsafe during breaks at school as well as of having experienced being threatened, hit or chased in the neighborhood the past 6 months, more often than those with shared residence.

**Table 5:** Working conditions and safety in school and in the neighborhood. Weighted percent and coefficients from linear probability models (LPM). *n* = 5055–5118

	Household with two custodial parents (ref.)	Shared residence	Household with one custodial parent	Sig. diff. Shared residence vs. One custodial parent
Too high pace at school^a^				
%	12.0	10.2	16.2	
Crude^b^	0.00	-0.01	0.03	n.s.
Adjusted^c^	0.00	-0.01	0.02	n.s.
Lack of order in classroom				
%	43.2	47.0	46.6	
Crude^b^	0.00	0.03	0.03	n.s.
Adjusted^c^	0.00	0.03	0.03	n.s.
Teachers help in school when needed				
%	93.8	92.3	92.8	
Crude^b^	0.00	-0.01	0.00	n.s.
Adjusted^c^	0.00	-0.02	0.00	n.s.
I do better at school than most others				
%	49.2	46.8	40.1	
Crude^b^	0.00	-0.03	-0.09***	n.s.
Adjusted^c^	0.00	-0.03	-0.08***	n.s.
Feel unsafe during breaks at school				
%	2.3	2.1	4.3	
Crude^b^	0.00	0.00	0.02**	**
Adjusted^c^	0.00	0.00	0.02**	*
Been threatened, hit or chased in my neighborhood				
%	6.3	5.9	11.4	
Crude^b^	0.00	0.00	0.06***	***
Adjusted^c^	0.00	0.00	0.05***	**
Feel unsafe in my neighborhood				
%	15.4	17.1	21.1	
Crude^b^	0.00	0.02	0.07***	n.s.
Adjusted^c^	0.00	0.03	0.06***	n.s.

^a^Question posed only during 2007–2008 (*n* = 2264)

^b^Adjusted for gender, age group, and survey year

^c^Adjusted for gender, age group, parental education, parental country of birth, and survey year

***p < 0.001 **p < 0.01 *p < 0.05

### Culture and Leisure Time Activities

With regard to culture and leisure time activities, the only significant difference between children living with two parents in one household and those with shared residence was found for housework, with children with shared residence being less likely to participate in housework at least 3 h per week, as presented in Table 6. Some differences were found between children living with two parents and those living with one parent. Children in the latter group were less likely to read books or participate in organized sport activities on a weekly basis, and to have visited the theater during the last six months. Children living with one parent compared to those in households with two custodial parents reported less often that they participated in organized non-sport activities on a weekly basis, although this association was attenuated and non-significant in the adjusted model. With regard to the differences between children with shared residence and those living with one parent, the former category was more likely to participate in sports activities and to have visited the theater and a museum during the last six months. In addition, children with shared residence were less likely than those living with one parent to have participated in housework at least 3 h per week, but this difference became non-significant in the adjusted model. Children living with one parent compared to those with shared residence reported less often going to the cinema during the last six months, but this difference also became non-significant in the adjusted model.

**Table 6:** Culture and leisure time activities. Weighted percent and coefficients from linear probability models (LPM). *n* = 5025–5123

	Household with two custodial parents (ref.)	Shared residence	Household with one custodial parent	Sig. diff. Shared residence vs. One custodial parent
Read books weekly				
%	57.8	55.5	48.8	
Crude^a^	0.00	-0.02	-0.07**	n.s.
Adjusted^b^	0.00	-0.01	-0.06**	n.s.
Organized sports activity weekly				
%	68.9	65.4	51.2	
Crude^a^	0.00	-0.03	-0.15***	***
Adjusted^b^	0.00	-0.03	-0.14***	***
Organized non-sport activity weekly				
%	23.0	20.4	17.1	
Crude^a^	0.00	-0.03	-0.04**	n.s.
Adjusted^b^	0.00	-0.03	-0.03	n.s.
Do housework ≥ 3 h weekly				
%	30.2	23.8	31.8	
Crude^a^	0.00	-0.06**	0.00	*
Adjusted^b^	0.00	-0.05*	-0.01	n.s.
Theater (last six months)				
%	27.2	27.2	23.7	
Crude^a^	0.00	0.01	-0.04*	*
Adjusted^b^	0.00	0.01	-0.04*	*
Cinema (last six months)				
%	78.7	81.5	78.6	
Crude^a^	0.00	0.03	-0.02	*
Adjusted^b^	0.00	0.03	-0.01	n.s.
Museum (last six months)				
%	35.3	39.1	32.7	
Crude^a^	0.00	0.05	-0.03	**
Adjusted^b^	0.00	0.04	-0.02	*

^a^Adjusted for gender, age group, and survey year

^b^Adjusted for gender, age group, parental education, parental country of birth, and survey year

***p < 0.001 **p < 0.01 *p < 0.05

## Discussion

This study of the living conditions among Swedish children in different family forms highlights the inequalities in social and material resources between the groups, despite controlling (at least partly) for possible selection bias. The results provide a general picture that many Swedish children have good access to material as well as social resources but, importantly, the resources are not evenly distributed. Interestingly, the living conditions for children in shared residence resembles that of children living with two custodial parents in one household, to a great extent, while the situation for children living with one custodial parent is worse for most of the studied outcomes compared with children who live with two parents in the same household. As an attempt to adjust for selection effects, the analyses were adjusted for parental education and parental country of birth, known to differ between the living arrangement groups (Juby et al. 2005; Kitterod and Lyngstad 2014). Yet, overall, the estimates for the identified differences were not affected much when adjusted for these characteristics. However, it is possible that there are other selection mechanisms for the different living arrangements for children that we have not been able to study here, for example, how well the parents get along and cooperate.

### Children with Separated Parents

This study focuses on children of separated parents, with shared residence or living with one custodial parent. Many previous studies have explored the differences between those children living with two custodial parents in one household compared with children who experienced family break-up, linking the experience of parental separation and the connected loss of resources and potential exposure to conflict and poorer outcomes in children with separated parents (Amato and Sobolewski 2001; Andress et al. 2006; Ängarne-Lindberg and Wadsby 2009). In the present study, however, children with separated parents were grouped after living arrangement. This grouping further elucidates the differences in children’s living conditions after parental dissolution between those with shared residence and those living with one custodial parent. When comparing these two categories with children living with two custodial parents in one household, only a small number of the indicators selected for this study differed between children with two custodial parents in the same household and those with shared residence, while a substantial majority was found to be worse for children living with one custodial parent. In this cross sectional study, no causal relationship could be determined, and despite the adjusted analyses there is a possibility that the findings reflect selection effects, i.e., there are unobserved differences between parents who end up having different arrangements after separation. Yet, whatever the reasons might be, the children living with one of the custodial parents are subjected to more ill-health as well as to having fewer resources, social as well as material and cultural. This is in accordance with the literature on children of single parents, e.g., (Jonsson and Östberg 2010; Mood and Jonsson 2015). In the present study, however, parents who re-married or re-partnered were also included in the group for living with one custodial parent.

### Shared Residence versus Living with One Custodial Parent

Between the two post-separation family groups, about half of the studied variables were shown to differ, spread across the study areas, indicating that children with shared residence tended to have more resources than those living with one custodial parent. This is in line with some previous studies looking at health differences between children with shared residence and in sole parental care in Sweden (Bergström et al. 2015, 2013; Fransson et al. 2015; Låftman et al. 2014) and in other parts of Europe (Vanassche et al. 2013; Westphal and Monden 2015). The results from the present study add to the existing literature by including a wider range of living conditions for children. The results indicate that the differences relate to health-related outcomes as well as economic and material conditions, relations with parents, experiences of safety, and access to cultural and leisure time activities. In contrast, differences in social relations with peers between children with shared residence and those living with one custodial parent were small and not significant after adjustment for parental education and country of birth. Few differences were also found in working conditions in school between children in the two post-separation living arrangements. Moreover, despite being worse off in the comparison, it should be emphasized that most of the children living with one custodial parent (around 80% or more) still reported getting along well with both mother and father.

### Children in Shared Residence – What about the Suggested Drawbacks?

In Sweden and elsewhere, the welfare of children with shared residence has been debated. Yet, the present study indicates that shared residence results in many benefits to households with two custodial parents, with both social and material resources being similar among children in these two types of living arrangements. Some exceptions were found, for instance, children with shared residence were more likely to report that they could not afford to join friends for activities, but they were also more likely to claim that their fathers had time for them. In an interview study with Swedish children with shared residence, some children experienced getting increased attention from each parent when seeing one parent at a time (Berman 2015). Exposure to parental conflict was not measured in Child-ULF, nor potential feelings of being emotionally torn between parents. Children with shared residence, however, reported more often getting along well with both parents than children living with one custodial parent. Moreover, we did not find any support for the suggested difficulties to maintain social contacts when moving between different neighborhoods (Prazen et al. 2011), as children with shared residence reported bringing friends home as well as visiting friends’ houses, in line with the other groups. This result may reflect the fact that Swedish parents tend to live rather close also after separation (Statistics Sweden 2015). Nonetheless, since children with shared residence were less likely to report that their parents knew most of their friends’ parents compared with their peers living with two custodial parents in the same household, this suggests a risk that these children might be less monitored or less supported. Children living only with one custodial parent however were also less likely to have parents who knew most of the friends’ parents. The present study also finds a difference in the reporting of the amount of housework, as children with shared residence were less likely to help out than children living with two custodial parents in the same household. The concerns regarding potential stress for children living in two homes (Gilmore 2006; McIntosh et al. 2011) was not apparent in the present study, but children living with one custodial parent were more likely to be stressed than those living with two custodial parents.

In sum, the results from this study show that children having two homes were well off in many of the areas studied. Importantly, though, children who did not live with two custodial parents were shown to be subjected to multiple disadvantages compared to the other groups. Thus, we might conclude that children whose parents chose shared residence for them are doing well overall. Noteworthy, however, since we have not been able to fully control for selection, one cannot draw the overall conclusion that children who live with one custodial parent would be better off with shared residence.

### Strengths and Limitations

The main contribution of the present study is that it adds to the knowledge base by reporting on everyday life conditions of children in different family forms, comparing children with shared residence with those living with two custodial parents in one household and those living with only one custodial parent. The data material is large and based on a nationally representative sample of adults in Sweden, including also the children who live in the adult respondents’ households. An advantage is that it combines information from children and parents. While children themselves should be the main informants of their own conditions, socioeconomic characteristics, to be as reliable as possible, are preferably measured through information derived from parents or from official registers (Jonsson and Östberg 2010). Indeed, parental education, in particular, has limited reliability when reported by adolescents (Looker 1989). Nevertheless, the data material also has some limitations. The non-response rate was relatively high among both adults and children. Furthermore, for children with separated parents, there was no information on the time point of the parental separation or on the length of the current living arrangement. Previous studies have shown that the time period surrounding the parental separation is, not surprisingly, the most difficult time for children (Hetherington and Stanley-Hagan 1999). Furthermore, the timing of the separation with regard to the age of the child could be of importance (Lansford et al. 2006), something that we lack information on. Other important aspects that were not measured include inter-parental conflict. Finally, the fact that the data are cross-sectional prevents us from making causal interpretations with support in the data and to follow children over time.

It should also be noted that not least because of the wide range of outcomes studied, it was beyond the scope of this paper to evaluate the possible mechanisms in the associations between children’s living arrangements and their living conditions. Hence, disentangling potential mechanisms and pathways between children’s living arrangements and various types of outcomes is a fruitful avenue for future research. Aspects relevant for future study may include socioeconomic factors such as household income, household social class, and working conditions of the parents, as well as psychosocial conditions such as parental wellbeing, stress, and conflict level between parents.

## Conclusion

The present study showed that children with shared residence largely tend to have living conditions on par with children who live with two custodial parents in the same household. In contrast, children living with only one custodial parent have poorer living conditions than their peers in households with two custodial parents and those with shared residence. This was particularly true for economic and material conditions, relations with parents, and health related outcomes, while fewer differences were found regarding school conditions (at least those studied here). The patterns remained robust and were only minimally affected when adjusting for parental education and country of birth. Future studies should address the potential mechanisms behind the poorer wellbeing among children living with one custodial parent compared with those in other living arrangements. With regard to inquiry on the living conditions of children with shared residence, a promising avenue for future research would be to apply a longitudinal approach in order to prospectively assess the role of timing of the parental separation and the relevance of selection effects, as well as to study the long-term consequences of growing up in different family forms.

## Appendix 1. Dependent Variables, Detailed Description

### Economic and Material Conditions

*Having an own room* was constructed from the question “Do you have any of the following?” and the item “A room of your own” The response categories were “Yes” and “No.” For children with shared residence, the response categories differed across the survey years. During 2007–2008, all respondents were asked whether or not they had their own room. During 2009–2011, children with shared residence were asked to specify whether they had their own room in their mother and father’s home, respectively. Of these, 19% answered that they had their own room in one home, and 79% in both homes. For the analyses, children who responded that they had their own room in either one or both parents’ homes were coded as having their own room.

*Cash margin* was constructed from the question “If you suddenly needed 100 SEK [about 10 euro] for tomorrow, e.g., to go to the movies, would you be able to get it? If you can get or if you have 100 SEK, you answer yes.” Those who replied “Yes” were coded as having cash margin, as opposed to those who replied “No” or “Don’t know.”

*Cannot buy same things as friends* was based on the question “Has there been an incidence where you were not able to buy something that you wanted, which many others of your age had, because you could not afford it? Think about the last six months.” Children who replied “Yes, several times” were classified as not being able to buy the same things as friends, as opposed to those who answered “Yes, once” or “Never.”

*Cannot afford to join friends* was based on the question “Has there been an incidence where you were not able to join your friends, because you could not afford it? Think about the last six months.” Those who replied “Yes, several times” were classified as not being able to buy the same things as friends, as opposed to those who answered “Yes, once” or “Never.”

### Social Relations with Parents and Peers

*Gets on well with mother/father* were based on the questions “How do you and your mother get on?” and “How do you and your father get on?” Those who replied that they got on “very well” or “‘rather well” were categorized as getting on well with their mother/father as opposed to those who replied “okay,” “rather poorly,” or “very poorly.”

*Mother/father has time for me* were constructed from the questions “Does your mother usually have time for you if you want to talk about or do something?” and “Does your father usually have time for you if you want to talk about or do something?” It was considered as yes when the child stated “Yes, always” or “Yes, often” as opposed to “No, not so often“ or “No, never.”

*My parents know most of my friends’ parents* was constructed from the question “Do your parents know the parents of your friends?” It was considered as yes when the child stated “Yes, all or most,” as opposed to “Yes, some” or “No, none” (of the friends’ parents).

*Has at least one close friend in class* was derived from the question “Do you have any close friends in your school class?” with the response categories “Yes, one,” “Yes, two,” “Yes, three or more,” and “No.” The first three categories were merged to measure that the respondent had at least one close friend in class.

*Brings friends home weekly* was constructed from the question “How many days during an ordinary week, thus from Monday to Sunday, do you engage in one of the following in your spare time?” and the statement “Have friends at my house.” Response categories were “Every day,” “Several days a week,” “One day a week,” “Less often,” and “Never.” The first three categories were dichotomized against the last two.

*Visits friends in their home weekly* was measured from the same question as above and the item “Go to a friend’s house.”

*Bullied at school* was based on four types of common bullying situations. The question was formulated accordingly: “How often do you experience the following things at school?” The items used were: “Other students accuse you of things you have not done or things you cannot help with,” “No one wants to be with you,” “Other students show they do not like you somehow, for example, by teasing you or whispering or joking about you,” and “One or more students hit you or hurt you in some way.” Response categories for each item were: “Almost every day,” “At least once a week,” “At least once a month,” “Once in a while,” and “Never.” Those who had experienced at least one type of bullying on a weekly basis or more often were classified as bullied at school.

*Bullied on the Internet* was constructed from the question “When you have been on the Internet, has there been an incidence where someone has written something to you or spread rumors about you which made you sad, angry or worried? Think about the last six months.” The response categories were “Yes, several times,” “Yes, once,” and “No.” The former two were dichotomized against the last. This question was posed only during 2009–2011.

### Health and Health Behaviors

*Self-rated health (less than good)* was created from the question “Do you think your health is…” with response categories “Very good,” “Good,” “Quite good,” and “Bad.” Those who had responded to any of the latter two categories were classified as reporting less than good self-rated health.

*Psychological complaints* were assessed through three statements: “I often feel sad or down,” “I’m often tense and nervous,” and “I’m often grouchy and irritated.” The response alternatives were “Matches exactly,” “Matches roughly,” “Matches poorly,” and “Does not match at all.” Those who replied “Matches exactly” or “Matches roughly” to at least two of the three statements were categorized as reporting psychological complaints.

*Psychosomatic complaints* were constructed from the question “During the past six months, how often have you had the following problems?” The items used were “Headache,” “Stomach-ache,” and “Difficulty falling asleep.” The response categories were “Everyday,” “Several times a week,” “Once a week,” “Some time during the month,” and “Less often or never.” Those who had reported at least two complaints at least weekly were classified as having somatic complaints.

*Stressed weekly* was measured from the question “During the past six months, how often have you had the following problems?” and the item “Felt stressed.” Children reporting stress more than once per week were categorized as suffering from stress.

*Smoking weekly* was measured from the question “During the last six months, how often did any of the following things happen?” and the item “You smoked.” The response categories were “Every day,” “Several times a week,” “Once a week,” “Some time during the month,” and “Less often or never.” Those who replied that it had happened weekly or more often were classified as smoking weekly. During 2007–2008, this question was posed to all respondents, but in 2009–2011 it was posed only to respondents aged 13–18 years.

*Alcohol use at least every other week* was constructed from the question “How often have you had alcohol during the last six months?” with the response categories “Every week,” “Every other week,” “Every month,” “More seldom,” and “Never.” Those who replied every week or every other week were categorized as using alcohol at least every other week. The question was posed only to respondents aged 13–18 years.

*Skipping breakfast weekly* was derived from the question “During the last six months, how often did any of the following things happen?” and the item “You skipped breakfast.” The response categories were “Every day,” “Several times a week,” “Once a week,” “Some time during the month,” and “Less often or never.” Those who replied that it had happened weekly or more often were classified as skipping breakfast weekly.

*Exercise weekly* was constructed from the same question as above and the item “You exercised so you became breathless or sweaty.”

### Working Conditions and Safety in School and in the Neighborhood

*Too high pace at school* was measured from the question “When it comes to schoolwork, do you think you can work at the pace that you want to?” The response categories were “Yes,” “No, I want to work faster,” and “No, I want to work more slowly.” Those who replied that they wanted to work more slowly were classified as reporting too high pace. This question was posed only during 2007–2008.

*Lack of order in classroom* was measured from the item “It is usually calm in the classroom during classes,” with the response categories “Yes” and “No.” The item was reversely coded.

*Teachers help in school when needed* was constructed from the question “When it comes to schoolwork, do you think you get the help you need from the teachers at school?” with the response categories “Yes, always” and “Yes, often” dichotomized against “No, not so often” and “No, never.”

*I do better at school than most others* was constructed from the question “Compared with your classmates, how good do you believe you are in school?” The response categories were “Among the best,” “Better than most others,” “About as good as most others,” “Less good than most others,” and “Among the least good.” Participants who answered any of the first two options were coded as responding that they did better at school than most others.

*Feeling unsafe during breaks at school* was constructed from the question “Do you feel safe during breaks at school?” with the response categories “Yes” and “No.”

*Been threatened, hit or chased in my neighborhood* was assessed by the question “There has been an incident where I was threatened, hit, or chased in the past 6 months” and the response alternatives “Yes” and “No.”

*Feeling unsafe in the neighborhood* was constructed from the two questions “Do you feel safe outside in your neighborhood during daytime” and the same question regarding “at night” and the response alternatives “Yes” and “No”. Those who responded “No” on either of the two questions were coded as feeling unsafe while those who responded “yes” on both was coded as feeling safe.

### Culture and Leisure Time Activities

*Read books weekly* was measured from the question “During a usual week how many days do you usually…” and the item “Read books”. The response categories were “Every day”, “Several days a week”, “One day a week”, “More seldom” and “Never”.

*Organized sports activity weekly* was measured from the same question as above and the item “Practice sports in a club, e.g., football, horse-riding, swimming”.

*Organized non-sport activity weekly* was measured from the same question as above and the item “Attend an activity with an adult leader which has not to do with sports, e.g. scouts, theater, chess”.

*Do housework ≥ 3 h weekly* was based on the question “About how many hours per week do you usually help out at home?” with the response categories “Less than 1 h”, “1–2 h”, “3–4 h”, “5 h or more” and “I usually don’t”. The response alternatives “3–4 h” and “5 h or more” were used to form the category at least 3 h as opposed to less.

*Theater* was based on the question “Did you visit any of the following during the past 6 months?” and the item “Theater”, with the response categories “Yes” and “No”.

*Cinema* was based on the same question as above and the item “Cinema”.

*Museum* was based on the same question as above and the item “Museum or art exhibition”.
